# Pulsed electroconversion for highly selective enantiomer synthesis

**DOI:** 10.1038/s41467-017-02190-z

**Published:** 2017-12-12

**Authors:** Chularat Wattanakit, Thittaya Yutthalekha, Sunpet Asssavapanumat, Veronique Lapeyre, Alexander Kuhn

**Affiliations:** 1 Department of Chemical and Biomolecular Engineering, School of Energy Science and Engineering, Vidyasirimedhi Institute of Science and Technology, Rayong, 21210 Thailand; 2 Univ. de Bordeaux, CNRS, ISM, UMR 5255, Bordeaux INP, Site ENSCBP, 16 Avenue Pey Berland, FR-33607 Pessac, France

## Abstract

Asymmetric synthesis of molecules is of crucial importance to obtain pure chiral compounds, which are of primary interest in many areas including medicine, biotechnology, and chemistry. Various methods have been used very successfully to increase the enantiomeric yield of reaction pathways, but there is still room for the development of alternative highly enantioselective reaction concepts, either as a scientific challenge of tremendous fundamental significance, or owing to the increasing demand for enantiopure products, e.g., in the pharmaceutical industry. In this context, we report here a strategy for the synthesis of chiral compounds, based on pulsed electrochemical conversion. We illustrate the approach with the stereospecific electroreduction of a prochiral model molecule at chiral mesoporous metal structures, resulting in an enantiomeric excess of over 90%. This change of paradigm opens up promising reaction schemes for the straightforward synthesis of high-added-value molecules.

## Abstract (web summary)

Synthesis of chiral molecules mostly relies on asymmetric catalysis. Here, the authors developed a pulsed electrochemical method to convert a prochiral ketone into the corresponding chiral alcohols with very high enantioselectivity on chiral-imprinted mesoporous platinum.

## Introduction

Molecular chirality is one of the most fascinating topics in chemical research due to its importance in many areas such as materials engineering^1–3^, surface science^4–8^, pharmaceutics^9^, separation science, and catalysis^10^. Therefore, the scientific community has made enormous efforts to obtain enantiomerically pure compounds, either based on the chiral pool^11–13^, resolution of racemates^14, 15^, or asymmetric synthesis^16^. Among them, enantioselective synthesis has been extensively developed as a controllable process^17–19^, and successfully achieved via catalysis based on the use of chiral coordination complexes^20, 21^, imprinted polymers^22, 23^, and biocatalysts^24, 25^. However, there are also potential drawbacks, such as too flexible molecular structures^26^, low thermal and chemical stability^18^, and tedious catalyst preparation^27^. Furthermore, some of the most successful strategies are based on homogeneous catalysis with inherent problems of catalyst separation from the reaction product^28, 29^. Therefore, heterogeneous reaction schemes may have significant advantages^30, 31^. For electrochemical reactions, electronic conductors are mandatory and in this context, the development of metals with chiral surfaces is an interesting choice to carry out heterogeneous enantioselective reactions.

Several concepts have been tested in the past such as the binding of chiral ligands to metal surfaces^32^, the adsorption of chiral molecules on surfaces to produce chiral metals^33^, or metal oxides^34, 35^, the cutting of high Miller index planes of metal monocrystals^36, 37^, and the distortion of symmetrical metal structures^38^. The design and synthesis of chiral metal structures has been explored for several of them (e.g., Pt, Pd, Ag, Au, Cu, Rh)^39–44^. Recently it has been reported that even the bulk of metals can be imprinted with chiral information^41, 45^. Our group demonstrated that chiral cavities can be generated in mesoporous platinum, based on the electrochemical reduction of platinum ions around a self-assembled liquid crystal phase of non-ionic surfactant and chiral template molecules^46^. These materials exhibit fascinating properties because they can be used to discriminate the enantiomers of an imprinted chiral molecule and other chiral molecules with analog structures^46–48^. The reason for the remarkable chiral recognition can be attributed to the fact that a large number of chiral cavities are located at the internal surface of the mesoporous network.

In addition, theses chiral-encoded mesoporous metal surfaces have also been shown to induce a certain degree of enantioselectivity during the electrochemical synthesis of mandelic acid from its prochiral precursor^47^, meaning that one of the possible stereoisomers of the molecule is formed preferentially. However, the enantiomeric excess is very modest with typical values in the few percent range. One reason for this low performance is that the external surface of the electrode is not imprinted with chiral information and therefore produces the final compound in a completely non-stereospecific way. Therefore, the product is a mixture of enantiomers produced in a quite selective way inside the mesoporous phase, and a racemate resulting from the reaction of precursor molecules at the outer surface. This intrinsically prevents from achieving high values of enantiomeric excess. A promising way to increase the ratio between precursor molecules that are transformed inside the metal matrix and those reacting at the outside is to play with the competition of transport and reaction kinetics. In this context, electrochemistry has the unique advantage of allowing a perfectly time-controlled triggering of the involved redox reactions^49^. The interplay between filling the chiral cavities with precursor molecules by diffusion and intermittently transforming them by applying an appropriate potential pulse constitutes an efficient heterogeneous reaction concept which is solely possible with electrochemistry.

In the present study, we describe an application of this concept to the highly selective asymmetric synthesis of chiral molecules based on the pulsed electroconversion of a prochiral molecule with chiral-imprinted mesoporous metal. This strategy is expected to avoid almost completely the interference of reactions at non-imprinted sites. In a proof-of principle synthesis, the two enantiomers of 1-phenylethanol (PE) are successfully and selectively synthesized via pulsed electroconversion of prochiral acetophenone on chiral-imprinted mesoporous platinum (CIMP). The degree of enantioselectivity can be tuned by changing the pulse time and the amount of imprinted chiral cavities.

## Results

### Concept of enantioselective pulsed electrosynthesis

**Fig. 1:**
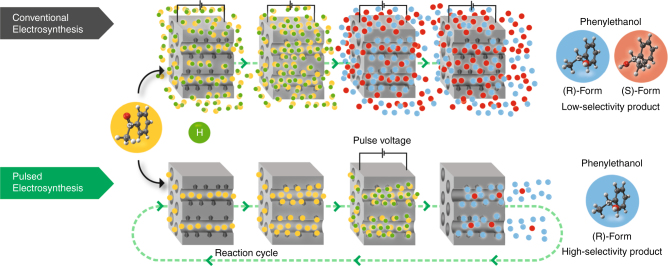
Comparison of conventional and pulsed chiral electrosynthesis. Illustration of the conversion of achiral acetophenone (yellow dots) into *(R)*- and *(S)*-PE (blue and red dots, respectively) by stereoselective addition of hydrogen (green dots) during conventional and pulsed asymmetric electrosynthesis using chiral-encoded metal electrodes

Conventional steady-state electroreduction of a prochiral molecule on chiral mesoporous platinum exhibits some enantioselectivity because the handedness of the product is influenced by the geometry of the chiral metal cavities^47^. However, the interference of concomitant reactions at non-imprinted metal located at the external electrode surface cannot be prevented and therefore leads to a decrease in overall stereoselectivity. Thus, in order to enhance the enantioselectivity, the major issue is to suppress such interferences. For this, we propose a concept allowing to improve the degree of enantioselectivity by using pulsed electrosynthesis. The differences between conventional and pulsed electroconversion of a prochiral starting molecule are illustrated in Fig. 1.

In the case of conventional electrosynthesis, carried out under steady-state conditions (i.e., constant applied potential), the achiral molecules (in this example acetophenone, symbolized by yellow dots) can combine with hydrogen (green dots) both, inside the chiral cavities and at the outermost surface of the electrode, resulting in low excess of either *(R)-* or *(S)*-PE (blue and red dots, respectively) as a function of the imprinted enantiomer. In strong contrast to this, pulsed electroconversion can lead to a very significant increase of the enantioselective properties based on the following mechanism. When no potential is applied, the prochiral educts diffuse into the mesopores and adsorb in the chiral cavities that are decorating the pore walls. Once the host matrix is filled, a potential pulse of appropriate amplitude and duration is applied to reduce the adsorbed precursor molecules in an enantioselective way. During the subsequent relaxation period at open circuit, the formed products can leave the matrix and, simultaneously, fresh precursor can enter the mesopores. Then the potential pulse is applied again. Repeating this cycle many times will lead to the gradual accumulation of the preferentially formed enantiomer in the bulk solution.

Obviously, during each potential pulse precursor molecules can also be reduced at the non-selective outer electrode surface. However, the quantity of converted molecules at the outer surface is much smaller because the internal surface area of mesoporous metals is typically two to three orders of magnitude higher compared to the external surface area^50^. This means that, depending on the pulse duration, the interference of electroreduction at the external surface can be almost completely suppressed, and results in the highly selective production of one enantiomer with respect to the other.

### Synthesis of chiral-encoded mesoporous platinum surfaces

**Fig. 2:**
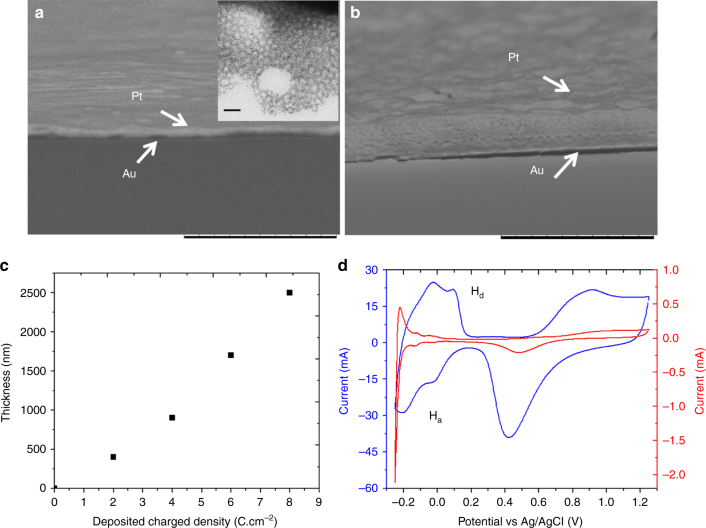
Structural characterization of chiral mesoporous platinum. **a**,** b** Scanning electron microscopy images of cross sections of mesoporous metal films obtained for injected charge densities of 2 and 8 C cm^−2^. Scale bar 10 μm. The inset in **a** is a TEM image of such a mesoprous structure with a scale bar of 20 nm. **c** Relationship between film thickness and the deposited charged density. **d** Cyclic voltammograms of flat platinum (red) and a chiral-imprinted mesoporous platinum film obtained by injecting a charge density of 8 C cm^−2^ (blue) recorded in 0.5 M H_2_SO_4_ at 100 mV s^−1^. H_a_ (hydrogen adsorption) and H_d_ (hydrogen desorption)

Chiral-encoded mesoporous platinum has been synthesized analog to previous reports^46–48^. In brief, the hexagonal (H_1_) structure of a lyotropic liquid crystal, obtained by the self-assembly of the surfactant Brij 56, has been used as a template to control the mesoporous structure of the metal^50^. The interaction between the hydrophilic outer part of the surfactant columns and the OH groups of the chiral template leads to an oriented geometry of chiral cavities when metal is grown around these supramolecular structures^46^. Several template molecules have been successfully used for controlling the shape of the chiral imprint, such as DOPA (3,4-dihydroxyphenylalanine)^46^, mandelic acid^47^, and PE (this work). CIMP films with different thickness have been prepared on gold-coated glass slides for the subsequent use as working electrodes in the experiments of pulsed electroconversion of the prochiral molecule (Fig. 2a, b). The morphology and the dimensions were uniform and similar to what has been described in previous reports^46, 47^. The mesoporosity is clearly visible when performing transmission electron microscopy (see inset of Fig. 2a). The film thickness is proportional to the injected charge density (Fig. 2c) and various thicknesses were used for tuning the enantioselectivity, because a more pronounced selectivity can be observed when increasing the film thickness^46^.

To characterize the active surface area related to the mesoporous feature, cyclic voltammetry of the porous platinum electrodes in 0.5 M of H_2_SO_4_ was used (Fig. 2d and Supplementary Figure 1). The peaks between −0.20 and 0.20 V can be attributed to the hydrogen adsorption and desorption, whereas the oxidation of Pt and the reduction of PtO_n_ occur at about 0.7 and 0.5 V, respectively. The significant enhancement of active surface area of a porous platinum film (blue) compared with flat platinum (red) is translated into a roughness factor of 33.4. No additional increase in active surface area is observed when comparing a chiral-imprinted and a non-imprinted mesoporous electrode (Supplementary Figure 1). This indicates that the high surface area is essentially due to the mesoporosity and not to the chiral cavities.

**Fig. 3:**
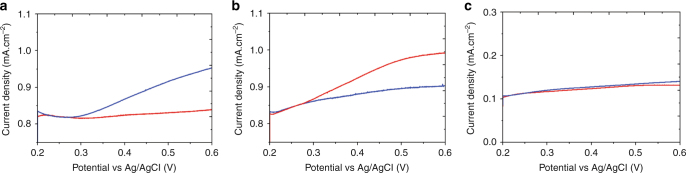
Electrochemical characterization of enantioselectivity. Differential pulse voltammetry (DPV) in 4 mM *(R)*-PE (blue), and *(S)-*PE (red), using 0.2 M KNO_3_ as supporting electrolyte with **a** a CIMP electrode imprinted with *(R)*-PE, **b** a CIMP electrode imprinted with *(S)*-PE, **c** a non-imprinted mesoporous platinum electrode. All electrodes were prepared using a deposition charge density of 2 C cm^−2^ and a PE/PtCl_6_^2−^ weight ratio of 0.05 for the imprinted ones

In order to verify the enantioselective properties of the so-obtained surfaces, the recognition of 1-PE via electrooxidation was monitored using differential pulse voltammetry as shown in Fig. 3a–c. The explored potential window has been limited on the positive side to +0.6 V in order to avoid interference with the oxidation of platinum. When comparing the different records, a more pronounced signal for *(R)*-PE with respect to *(S)*-PE can be observed in the case of chiral mesoporous platinum imprinted by *(R)*-PE (Fig. 3a). In contrast, the electrode imprinted by the opposite enantiomer exhibits a higher amplitude for *(S)*-PE with respect to *(R)*-PE (Fig. 3b). It is necessary to confirm that the different signals for the two enantiomers are not artifacts; in particular they might be due to chiral template molecules remaining inside the metal after the imprinting. Therefore, the removal of the template molecules was monitored by measuring the amount of chiral molecules leaving the metal phase as a function of rinsing time using UV-Vis spectroscopy (see Supplementary Figure 2). It clearly demonstrates that no more template molecules are leaving the cavities after sufficient rinsing. As expected, no enantioselective discrimination properties are revealed for non-imprinted mesoporous platinum (Fig. 3c), meaning that no chiral information is encoded without using a chiral template molecule in the preparation mixture. These observations confirm that CIMP can be successfully prepared using this general approach with various types of a chiral templates and the so-obtained host matrix exhibits intrinsic chiral properties even after removable of the chiral template.

### Pulsed enantioselective synthesis of chiral phenylethanol

**Fig. 4:**
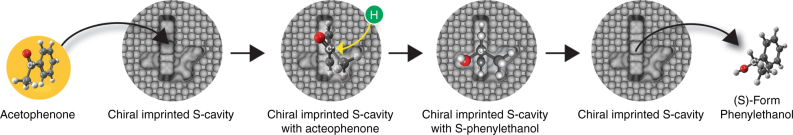
Enantioselective synthesis steps. Illustration of the electrosynthesis of *(S)*-phenylethanol from acetophenone based on its adsorption in chiral-imprinted cavities of the mesoporous metal, followed by its stereoselective reduction and desorption

The enantioselective synthesis of PE from acetophenone has been chosen as a model system because it is an interesting compound in bio-oil in which ketones are present in a large portion (27 wt%)^51^. Acetophenone is a very simple aromatic ketone, and therefore well-suited for first proof-of-principle studies, which then might be extended to other, more complex, ketones^52, 53^. In addition, the conversion of this compound into a pure enantiomer of 1-PE as an important chiral building block in pharmaceutical industries can be considered as a representative example for the production of high-added-value products. The molecule is going to adsorb inside the chiral cavities present in the mesoporous structure and this will direct the synthesis preferentially towards one of the two enantiomers of phenylethanol, depending on which one has been imprinted (Fig. 4). After the reduction is accomplished, the product is desorbed and the cavity is again available for the transformation of further molecules.

In order to test the described concept and optimize the different electrosynthesis parameters, such as the reduction potential, the thickness of the Pt film and the amount of imprinted chiral sites, the conventional steady-state electroreduction of acetophenone was first studied. To prevent the reduction of protons at too negative potentials, the potential has been fixed in the range of −0.40 to −0.50 V, because the carbonyl function of acetophenone starts to reduce at these potentials (see Supplementary Figure 3). Supplementary Table 1 summarizes the relationship between the enantiomeric excess and the reduction potential, the amount of chiral template, and the metal layer thickness. A more positive reduction potential can allow a slight increase of enantiomeric excess (Entries 1 and 2 in Supplementary Table 1). Increasing the weight ratio of chiral template to PtCl_6_^2−^ from 0.05 to 0.15, improves the enantiomeric excess by a factor of about two (Entries 2–4 in Supplementary Table 1). A similar tendency can also be observed when using electrodes imprinted with the opposite enantiomer (Entries 5 and 6 in Supplementary Table 1). This confirms the presence of a pronounced chiral character of the metal surfaces, able to promote the transformation of a prochiral molecule into a chiral compound. As stated above, the degree of enantioselectivity is correlated with the thickness of the porous metal layer. Entries 5 and 6 in Supplementary Table 1 indicate a significant improvement of enantiomeric excess when increasing the amount of deposited metal. From these preliminary optimization experiments it is possible to identify as the best conditions for a maximized enantiomeric excess a reduction potential of −0.45 V, a PE enantiomer/PtCl_6_^2−^ weight ratio of 0.15, and a metal deposition charge density of 8 C cm^−2^.

**Fig. 5:**
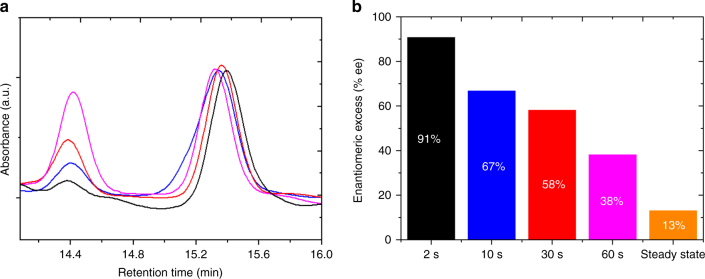
Monitoring of enantioselectivity as a function of pulse time. **a** HPLC chromatograms of the electrosynthesis products obtained with an electrode imprinted with *(S)*-PE (*(S)*-PE/PtCl_6_^2−^ weight ratio of 0.15) at −0.45 V for different pulse times; 60 s pulse time (pink), 30 s (red), 10 s (blue) and 2 s (black), with 120 s relaxation time in all cases. The peaks at retention times of 14.4 and 15.3 min belong to *(R)*-PE and *(S)*-PE, respectively; **b** Histogram of the relationship between enantiomeric excess and pulse time using the same color code and showing for comparison also the value obtained in a steady-state experiment (orange)

These conditions were maintained identical for studying the impact of a pulsed driving force on enantioselectivity. As can be seen in Fig. 5a, pulsing the potential has a tremendous impact of the experimental outcome. The high-performance liquid chromatography (HPLC) records obtained with a chiral stationary phase column, which have been normalized with respect to the concentration of *(S)*-PE, clearly show that the electroreduction of acetophenone on chiral mesoporous platinum imprinted with *(S)*-PE produces *(S)*-PE with extremely high selectivity for the shortest pulses. This tendency is quantitatively summarized in Fig. 5b. For long pulses, the unspecific contribution from the acetophenone reduction at the non-chiral outer electrode surface has a strong influence and consequently leads to modest enantiomeric excess. However, at a very short pulse time (2 s) the %ee is clearly dominated by the conversion occurring almost exclusively inside the mesoporous matrix, owing to its large internal surface area when compared with the area of the non-imprinted outer surface. In this extreme case it reaches values slightly above 90%. In all cases, the relaxation time has been chosen long enough (120 s) to allow the product to leave the metal phase by diffusion and to refill it with fresh prochiral precursor before applying again the potential. This relaxation period has been overestimated on purpose to be on the safe side, and might be easily decreased by an order of magnitude when considering classic diffusion constants. This strategy leads to an almost complete suppression of the non-selective reaction at the external surface.

This effect can be understood by analyzing more closely the chronoamperometric curves obtained during such potential pulses (Supplementary Figure 4). The experiment consists of recording a chronoamperogram with an imprinted electrode when the potential is applied immediately after immersing it in a solution containing acetophenone. In this case acetophenone is not present in the mesopores and only reacts at the outer surface producing I_Out_ as a current (Supplementary Figure 4a, red curve). In a second experiment, the same electrode is allowed to equilibrate with the solution before applying the potential. Therefore, acetophenone will be present also in the mesoporous phase and lead to a current enhancement measured as I_Out+In_ (Supplementary Figure 4a, blue curve). Plotting the ratio between these two currents (I_(In+Out)_/I_(Out)_) gives a good indication of the relative contribution of the conversion of molecules inside the porous phase compared with the global amount of reacting molecules (Supplementary Figure 4b). It becomes evident that for rather short times (<2 s) the current is strongly influenced by molecules reacting inside the pores in a chiral environment, whereas for longer time intervals the overall current is mostly owing to molecules converted at the outer surface, which can be more easily resupplied by diffusion, but react in a non-enantioselective way. This leads to a decrease of %ee when increasing the pulse duration. These measurements demonstrate that the characteristic time scale of diffusion from the outside into the pores is much longer than that for the electron transfer rate inside the pores. A pulse time of 2 s seems therefore to be close to optimal, because for shorter pulses not all the molecules in the pores are converted, so the yield is lower, whereas for longer pulse times the outer electrode surface starts to interfere more and more and lowers the enantiomeric excess.

**Fig. 6:**
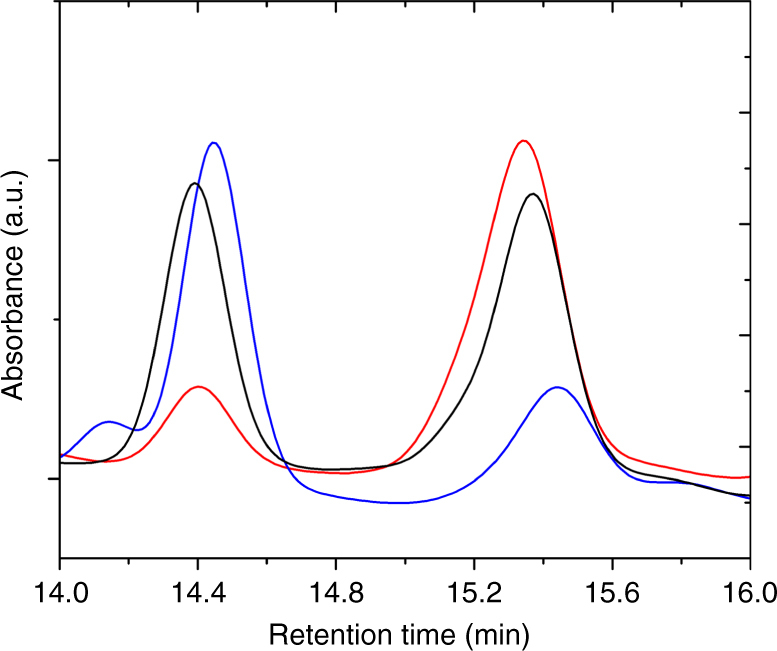
Monitoring of enantioselectivity as a function of imprinted enantiomer. HPLC chromatograms of the electrosynthesis products obtained by an electrode imprinted with *(S)*-PE (red) and an electrode imprinted with *(R)*-PE (blue) (PE/PtCl_6_^2−^ weight ratio of 0.15). The respective signals are compared with a racemate of PE that is obtained by electrosynthesis with an electrode imprinted with racemic PE (black). Peaks at retention times of 14.4 and 15.3 min belong to *(R)*-PE and *(S)*-PE, respectively

An opposite selectivity is observed when using electrodes imprinted with *(R)*-PE (pulse time of 10 s). The *(R)*-PE peak area is much higher than the *(S)*-PE peak (Fig. 6 and Supplementary Table 2). In addition, the enantiomeric excess obtained by using the electrodes imprinted with different chiral configurations was also confirmed by the results of circular dichroism measurements (Supplementary Figure 5) of the product solutions. Opposite signals in the circular dichroism spectra were obtained for electrodes imprinted with different enantiomers. The %ee values for electrodes imprinted with opposite enantiomers are in good agreement (+60.4 and −66.9% for imprinting with *(R)*-PE and *(S)*-PE, respectively), indicating the reproducibility of the electrodes.

One important aspect of heterogeneous reactions is the reusability of the active surface. We therefore studied the stability of the retained chiral information by measuring the degree of enantioselectivity of one and the same imprinted electrode in three consecutive experiments (Supplementary Figure 6 and Supplementary Table 3). The enantioselectivity is decreasing, but even after the third run %ee values are still very significant (about 40%). Obviously, such a decrease should be kept at a minimum and might be due to a rearrangement of the chiral cavities as a consequence of the surface mobility of the metal atoms. One possibility to stabilize the structural chiral information might be to perform the synthesis at lower temperatures.

Theoretically, the amount of generated chiral metal cavities should correlate with the content of chiral template molecule in the plating mixture. Therefore, we also examined the enantioselectivity as a function of the weight ratio of chiral template/PtCl_6_^2−^. It was found that the %ee dramatically improves from 12.0 to 66.9 % when increasing the chiral template to platinum ratio from 0.05 to 0.15 for a 10 s pulse time (Supplementary Figure 7a,b). However, the %ee starts to decrease when using a too high amount of chiral template. This may be explained by the fact that the self-assembly of the lyotropic liquid crystal phase is disturbed in the presence of too much chiral template, thus preventing the formation of the mesopores. Such a morphological change of the supramolecular template leads to less efficient imprinting of chiral information.

## Discussion

Highly stereoselective reduction of acetophenone with CIMP electrodes has been successfully achieved based on potential pulsed electrolysis. In conventional electrosynthesis a constant potential is applied to the working electrode. This leads to a steady-state conversion of molecules preferentially at the outer electrode surface, because diffusion into the mesopores is rather slow. As all parts of the platinum film are used as working electrode, but the chiral character is only present inside the mesopores, and not at the outer surface, the obtained enantiomeric excess is dominated by the unspecific reaction path. This eventually leads to almost a racemate even though selectivity can be slightly increased by changing the amount of chiral template in the plating mixture, the porous metal film thickness, and the reduction potential.

In order to circumvent this problem of modest stereoselectivity it is necessary to play with the competition between molecular transport efficiency into the enantioselective mesopores and reaction kinetics at the non-selective outer surface. The main strategy of this work has been to use repeatedly short potential pulses alternating with relaxation periods at open circuit. During the relaxation period the mesoporous matrix is filled with prochiral starting molecules by diffusion, which adsorb in the metal cavities bearing the chiral information. Application of a potential pulse leads to the conversion of these molecules inside the mesopores, but also of molecules at the external surface. The relative contribution of these two populations is tunable by the pulse length. To confirm this hypothesis, electroreduction experiments were carried out with varying pulse times. For long pulses the molecules in the matrix are converted immediately, but then cannot be resupplied efficiently enough during the remaining time of the pulse. For short pulses the situation is opposite, because the quantity of molecules stored and converted in the mesopores is much higher due to the very high internal surface area. Once this conversion has taken place, the potential is switched off so that the contribution of the much smaller outer surface area is almost negligible.

Interestingly, theses electrodes can be reused for several independent experiments and still show significant enantioselectivity. This demonstrates that the observed chiral features don’t originate from chiral template molecules left over in the porous structure after the initial metal electrodeposition step. Optimization of all different parameters finally allows reaching a %ee of over 90 %, which is extremely high for such a heterogeneous reaction scheme and starts to be competitive with respect to more classic approaches. However, the global conversion yield for a given reaction time is still modest (Supplementary Figure 8) owing to the exagerated relaxation time, but this can be further optimized in forthcoming studies. It is also important to mention that no significant quantities of side products, for example, owing to dimerization, seem to be formed as can be judged from HPLC analysis (Supplementary Figure 9). Thus, this opens up interesting perspectives in the frame of the development of selective chiral synthesis strategies for high-added-value products, one of the key challenges in modern organic chemistry.

## Methods

### Chemicals

Hexachloroplatinic acid hydrate (H_2_PtCl_6_. xH_2_O), polyoxyethylene (10) cetyl ether (Brij 56) together with *(R)*- and *(S)*-phenylethanol (PE) and MilliQ water were used to prepare the liquid crystal plating mixtures. Acetophenone was used as the prochiral reactant. Ammonium chloride was used as a supporting electrolyte. All chemicals were purchased from Sigma-Aldrich and used without further purification.

### Synthesis and characterization of CIMP

CIMP was prepared following a modified literature procedure^46^. Gold-coated glass slides (1 cm^2^) were used as substrates and PE enantiomer was used as a chiral template molecule. The liquid crystal mixture composed of 42 wt% of non-ionic surfactant (Brij 56), 29 wt% of chloroplatinic acid, 29 wt% of MiliQ water and the calculated amount of the chiral template molecule, was mixed until becoming completely homogeneous. The obtained, very viscous, paste was then placed as a several millimeter thick layer on a cleaned gold-coated glass slide. After insertion of a reference (Ag/AgCl) and counter electrode (platinum grid) in the liquid crystal paste, platinum was deposited at a constant potential of −0.1 V while measuring and integrating the current. Depending on the desired final thickness of the mesoporous metal layer, the injected charge densities have been varied from 2 to 8 C.cm^−2^. Subsequently, in order to remove the template molecules (surfactant and enantiomer), the electrodes were rinsed and soaked in MilliQ water for 24 h. Series of UV-Vis spectra of the different washing solutions were recorded to monitor the template removal as a function of rinsing time (Supplementary Figure 2).

The surface morphology and the thickness of the Pt film was characterized with a Hitachi TM-1000 tabletop electron microscope. The mesoporous nature of the deposit was studied by transmission electron microscopy (JEOL JEM-2010). In order to perform these TEM measurements, the platinum covered Au-coated glass slides were immersed in an aqueous solution of 4 wt% KI and 1 wt% I_2_ for ~20 min so that the underlying Au layer gets etched away. The mesoporous platinum film was then carefully detached from the electrode by slow immersion of the sample into DI water. The obtained floating film was finally transferred onto a TEM grid.

The electroactive surface area of CIMP was calculated from the integral of the hydrogen adsorption and desorption peaks obtained from the cyclic voltammograms of the Pt electrodes in 0.5 M H_2_SO_4_ recorded at a scan rate of 100 mVs^−1^ in the range from −0.25 to 1.25 V. The real surface area of Pt was estimated based on a calibration factor of 210 mC.cm^−2^ ^54^.

### Pulsed electroconversion of acetophenone on CIMP

In order to optimize the experimental parameters, including the reduction potential, the amount of chiral template used in the plating mixture, and the thickness of the platinum film, conventional steady-state electrochemical reduction of acetophenone on CIMP was performed with potentials ranging from −0.40 to −0.50 V for 13 h in a stirred aqueous solution of 10 mM acetophenone and 1 M ammonium chloride as supporting electrolyte. Before starting the reaction, the system was kept for 30 min in the mixture to let the solution diffuse inside the porous network. All experiments were carried out using an Autolab PGSTAT204 equipped with Ag/AgCl (sat. KCl), a Pt mesh, and CIMP as reference, counter, and working electrodes, respectively.

In the case of pulsed electrochemical reduction, the applied potential was −0.45 V with pulse times varying from 2 to 60 s. The relaxation time was kept constant at 120 s after each voltage pulse.

Finally, the products were extracted using heptane as solvent and the organic solution was analyzed by HPLC. HPLC was performed on a Shimadzu LC-2030C3D equipped with a chiral HPLC column (CHIRALPAK IB, 250 × 4.6 mm inner diameter) using a mobile phase containing 95% heptane/5% i-propanol at a flow rate of 0.5 ml min^−1^ and an optical detection at 215 nm. The integrated peak area was measured using Lab Solution software. To calculate the total conversion of acetophenone into product, a calibration curve of product was recorded (Supplementary Figure 8). To further confirm the enantiomeric excess, circular dichroism spectroscopy was performed on a Jasco J-815 spectrometer and circular dichroism spectra were recorded at a scan rate of 50 nm min^−1^ (Supplementary Figure 5).

### Data availability

All relevant data that support the current findings and which are not included in the main manuscript or the Supplementary Information are available from the corresponding authors on request.

## Electronic supplementary material

Supplementary Information

## References

[1] Gautier C, Bürgi T (2009). Chiral gold nanoparticles. ChemPhysChem.

[2] Chen W (2009). Nanoparticle superstructures made by polymerase chain reaction: Collective interactions of nanoparticles and a new principle for chiral materials. Nano. Lett..

[3] Liang J, Wu Y, Deng X, Deng J (2015). Optically active porous materials constructed by chirally helical substituted polyacetylene through a high internal phase emulsion approach and the application in enantioselective crystallization. ACS Macro Lett..

[4] Horvath JD, Koritnik A, Kamakoti P, Sholl DS, Gellman AJ (2004). Enantioselective separation on a naturally chiral surface. J. Am. Chem. Soc..

[5] Balents L, Fisher MPA (1996). Chiral surface states in the bulk quantum hall effect. Phys. Rev. Lett..

[6] Gellman AJ (2010). Chiral surfaces: accomplishments and challenges. ACS Nano..

[7] Arnaboldi S (2015). Inherently chiral electrodes: the tool for chiral voltammetry. Chem. Sci..

[8] Peng C, Lavrentovich OD (2015). Chirality amplification and detection by tactoids of lyotropic chromonic liquid crystals. Soft Matter.

[9] Farina V, Reeves JT, Senanayake CH, Song JJ (2006). Asymmetric synthesis of active pharmaceutical ingredients. Chem. Rev..

[10] Blaser HU, Pugin B, Spindler F (2005). Progress in enantioselective catalysis assessed from an industrial point of view. J. Mol. Catal. A.

[11] Blaser HU (1992). The chiral pool as a source of enantioselective catalysts and auxiliaries. Chem. Rev..

[12] Casiraghi G, Zanardi F, Rassu G, Spanu P (1995). Stereoselective approaches to bioactive carbohydrates and alkaloids with a focus on recent syntheses drawing from the chiral pool. Chem. Rev..

[13] Nugent WA, RajanBabu TV, Burk MJ (1993). Beyond nature’s chiral pool: enantioselective catalysis in industry. Science.

[14] Maier NM, Franco P, Lindner W (2001). Separation of enantiomers: needs, challenges, perspectives. J. Chromatogr. A..

[15] Okamoto Y, Ikai T (2008). Chiral HPLC for efficient resolution of enantiomers. Chem. Soc. Rev..

[16] Noyori R (2003). Asymmetric catalysis: Science and opportunities (Nobel lecture 2001). Adv. Synth. Catal..

[17] Heitbaum M, Glorius F, Escher I (2006). Asymmetric heterogeneous catalysis. Angew. Chem. Int. Ed..

[18] Cornils B, Herrmann WA (2003). Concepts in homogeneous catalysis: the industrial view. J. Catal..

[19] Yoon TP, Jacobsen EN (2003). Privileged chiral catalysts. Science.

[20] Lu Z, Ma S (2008). Metal-catalyzed enantioselective allylation in asymmetric synthesis. Angew. Chem. Int. Ed..

[21] Morris RH (2009). Asymmetric hydrogenation, transfer hydrogenation and hydrosilylation of ketones catalyzed by iron complexes. Chem. Soc. Rev..

[22] Kupai J, Rojik E, Huszthy P, Szekely G (2015). Role of chirality and macroring in imprinted polymers with enantiodiscriminative power. ACS Appl. Mater. Interfaces.

[23] Dai C (2014). Chiral sensing of Eu(III)-containing achiral polymer complex from chiral amino acids coordination induction. J. Polym. Sci. Part A.

[24] Savile CK (2010). Biocatalytic asymmetric synthesis of chiral amines from ketones applied to sitagliptin manufacture. Science.

[25] Matsuda T, Yamanaka R, Nakamura K (2009). Recent progress in biocatalysis for asymmetric oxidation and reduction. Tetrahedron.

[26] Verendel JJ (2010). Highly flexible synthesis of chiral azacycles via iridium-catalyzed hydrogenation. J. Am. Chem. Soc..

[27] Xia QH, Ge HQ, Ye CP, Liu ZM, Su KX (2005). Advances in homogeneous and heterogeneous catalytic asymmetric epoxidation. Chem. Rev..

[28] Funk TW, Berlin JM, Grubbs RH (2006). Highly active chiral ruthenium catalysts for asymmetric ring-closing olefin metathesis. J. Am. Chem. Soc..

[29] Noyori R, Ohkuma T (2001). Asymmetric catalysis by architectural and functional molecular engineering: Practical chemo- and stereoselective hydrogenation of ketones. Angew. Chem. Int. Ed..

[30] Song F, Wang C, Falkowski JM, Ma L, Lin W (2010). Isoreticular chiral metal−organic frameworks for asymmetric alkene epoxidation: Tuning catalytic activity by controlling framework catenation and varying open channel sizes. J. Am. Chem. Soc..

[31] Jeong KS (2011). Asymmetric catalytic reactions by NbO-type chiral metal-organic frameworks. Chem. Sci..

[32] Gross E (2013). Asymmetric catalysis at the mesoscale: gold nanoclusters embedded in chiral self-assembled monolayer as heterogeneous catalyst for asymmetric reactions. J. Am. Chem. Soc..

[33] Lawton TJ (2013). Long range chiral imprinting of Cu(110) by tartaric acid. J. Phys. Chem. C.

[34] Switzer JA, Kothari HM, Poizot P, Nakanishi S, Bohannan EW (2003). Enantiospecific electrodeposition of a chiral catalyst. Nature.

[35] Chen C, Shi H, Zhao G (2014). Chiral recognition and enantioselective photoelectrochemical oxidation toward amino acids on single-crystalline ZnO. J. Phys. Chem. C.

[36] Attard GA (2001). Electrochemical studies of enantioselectivity at chiral metal surfaces. J. Phys. Chem. B.

[37] Hazzazi OA, Attard GA, Wells PB (2004). Molecular recognition in adsorption and electro-oxidation at chiral platinum surfaces. J. Mol. Catal. A.

[38] Garzón IL (2003). Chirality, defects, and disorder ingold clusters. Eur. Phys. J. D.

[39] Farrag M (2016). Enantioselective silver nanoclusters: preparation, characterization and photoluminescence spectroscopy. Mater. Chem. Phys..

[40] Yan J (2016). Asymmetric synthesis of chiral bimetallic [Ag_28_Cu_12_(SR)_24_]_4_–nanoclusters via ion pairing. J. Am. Chem. Soc..

[41] Durán Pachón L (2009). Chiral imprinting of palladium with cinchona alkaloids. Nat. Chem..

[42] Yasukawa T, Suzuki A, Miyamura H, Nishino K, Kobayashi S (2015). Chiral metal nanoparticle systems as heterogeneous catalysts beyond homogeneous metal complex catalysts for asymmetric addition of arylboronic acids to α,β-unsaturated carbonyl compounds. J. Am. Chem. Soc..

[43] Zhan P, Wang ZG, Li N, Ding B (2015). Engineering gold nanoparticles with DNA ligands for selective catalytic oxidation of chiral substrates. ACS Catal..

[44] Fang Y (2017). Chiral sensing with mesoporous Pd@Pt nanoparticles. ChemElectroChem.

[45] Behar-Levy H, Neumann O, Naaman R, Avnir D (2007). Chirality induction in bulk gold and silver. Adv. Mater..

[46] Wattanakit C (2014). Enantioselective recognition at mesoporous chiral metal surfaces. Nat. Commun..

[47] Yutthalekha T (2016). Asymmetric synthesis using chiral-encoded metal. Nat. Commun..

[48] Yutthalekha T, Warakulwit C, Limtrakul J, Kuhn A (2015). Enantioselective recognition of DOPA by mesoporous platinum imprinted with mandelic acid. Electroanalysis.

[49] Yoshida JI, Kataoka K, Horcajada R, Nagaki A (2008). Modern strategies in electroorganic synthesis. Chem. Rev..

[50] Attard GS (1997). Mesoporous platinum films from lyotropic liquid crystalline phases. Science.

[51] Valle B, Gayubo AG, Aguayo AT, Olazar M, Bilbao J (2010). Selective production of aromatics by crude bio-oil valorization with a nickel-modified HZSM-5 zeolite catalyst. Energy Fuels.

[52] Undri A (2015). A simple procedure for chromatographic analysis of bio-oils from pyrolysis. J. Anal. Appl. Pyrolysis.

[53] González C, Marín P, Díez FV, Ordóñez S (2015). Hydrodeoxygenation of acetophenone over supported precious metal catalysts at mild conditions: Process optimization and reaction kinetics. Energy Fuels.

[54] Trasatti S, Petrii OA (1992). Real surface area measurements in electrochemistry. J. Electroanal. Chem..

