# Supplementary material for: Pulsed electroconversion for highly selective enantiomer synthesis
Source: Nat Commun. 2017 Dec 12;8:2087. doi: 10.1038/s41467-017-02190-z (PMC5727193; doi:10.1038/s41467-017-02190-z)
Supplement: Supplementary file 1 — Supplementary Information [file 41467_2017_2190_MOESM1_ESM.pdf]

Supplementary Figures

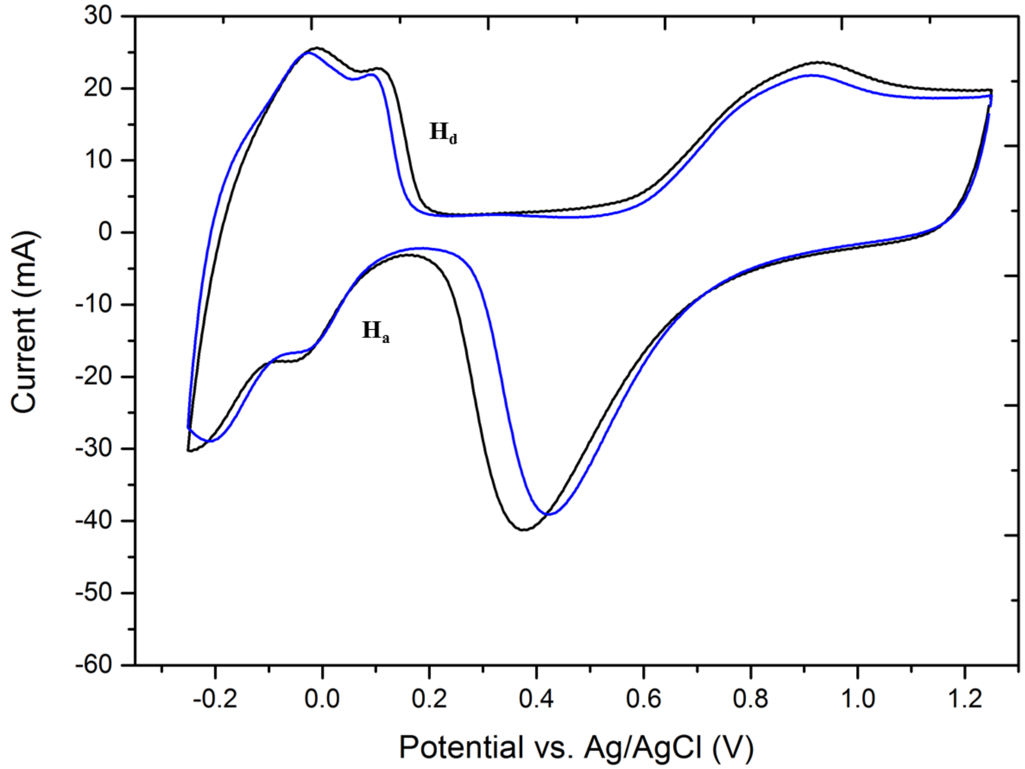

Supplementary Figure 1. Cyclic voltammograms of non-imprinted mesoporous platinum (blue) and a chiral-imprinted mesoporous platinum film (black) obtained by injecting a charge density of 8 C.cm^{-2} recorded in 0.5 M H_2SO_4 at 100 mV.s^{-1}. H_a (hydrogen adsorption) and H_d (hydrogen desorption).

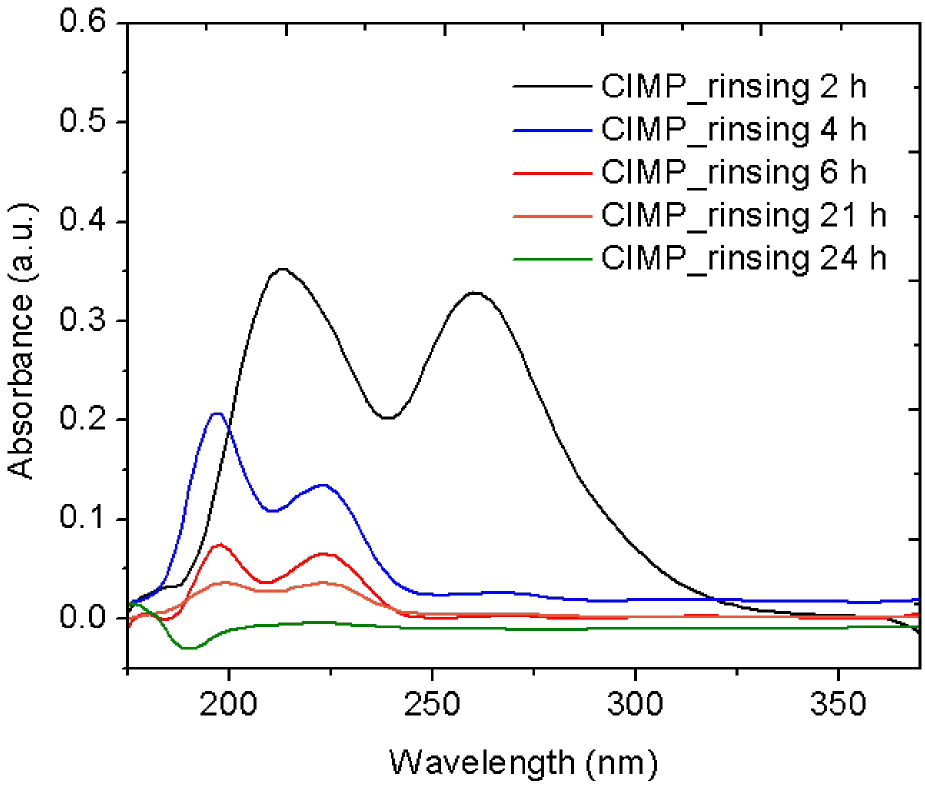

Supplementary Figure 2. Spectroscopic control of template removal. Series of UV-Vis spectra recorded with the different washing solutions obtained during template removal from a chiral mesoporous platinum electrode (CIMP) imprinted with *(R)*-PE using a *(R)*-PE/$PtCl_6^{2-}$ molar ratio of 0.05.

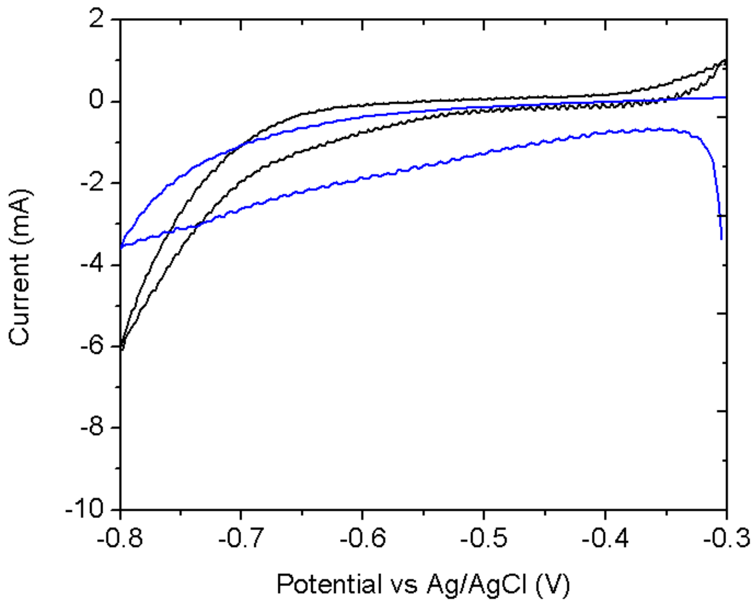

Supplementary Figure 3. Electroactive potential domain of acetophenone. Cyclic voltammograms of a normal (non-porous) platinum electrode in 1M NH_4Cl (black) and in NH_4Cl containing 43 mM acetophenone (blue) at a scan rate of 100 mVs^{-1}, showing reduction of the prochiral C=O group in the range from -400 to -700 mV.

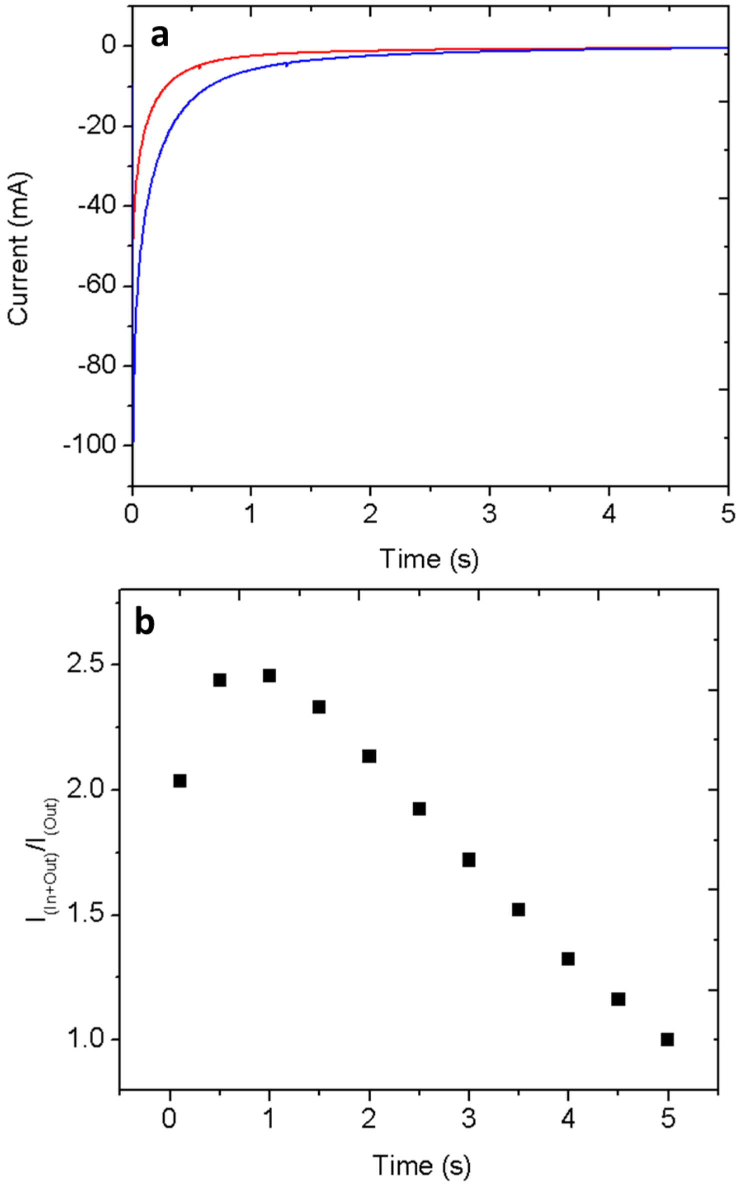

Supplementary Figure 4. Relative contribution of internal and external surface to the enantiomeric excess. (a) Chronoamperometric curve during a pulse electrosynthesis in 10 mM acetophenone using mesoporous platinum imprinted with (*S*)-PE/$PtCl_6^{2-}$ (weight ratio of 0.15) (reduction potential -0.45 V). Acetophenone is only reacting at the external surface of the electrode because the pores are empty (red, $I_{(Out)}$); acetophenone is reacting inside the porous structure and at the external surface of the electrode (blue, $I_{(In+Out)}$); (b) ratio of both currents as a function of electroreduction time, representing the relative contribution of molecules inside and outside the mesopores to the global current.

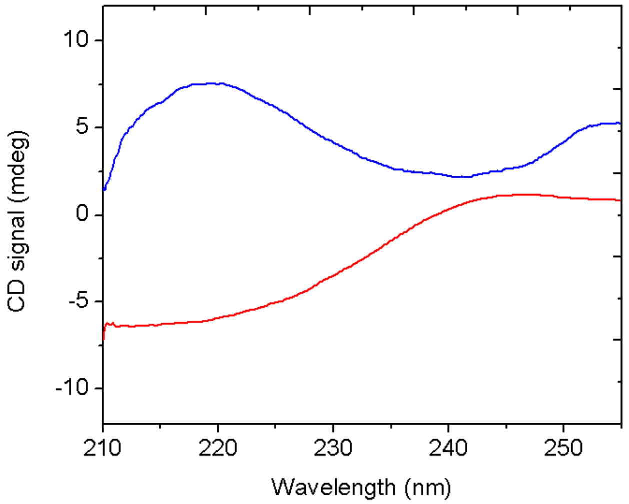

Supplementary Figure 5. Circular dichroism. CD spectra of the reaction mixture obtained from the enantioselective synthesis of 1-phenylethanol (10 mM) using the chiral mesoporous platinum imprinted with (*R)*-PE (blue) and (*S)*-PE (red) with a PE/$PtCl_6^{2-}$ weight ratio of 0.15 for pulsed electroreduction of acetophenone (reduction potential -0.45 V, relaxation time 120 s, pulse time 5 s).

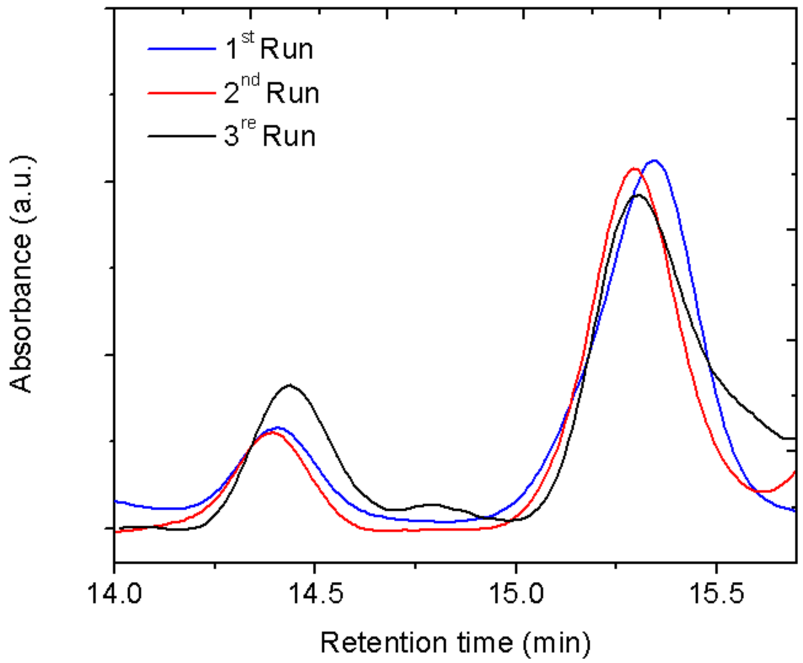

Supplementary Figure 6. Monitoring the stability of an imprinted electrode. HPLC chromatograms of the electrosynthesis products obtained by using the same electrode several times. (*S*)-PE/$PtCl_6^{2-}$ weight ratio of 0.15, at -0.45 V and a pulse time of 10s; 1st run (blue), 2nd run (red), and 3rd run (black).

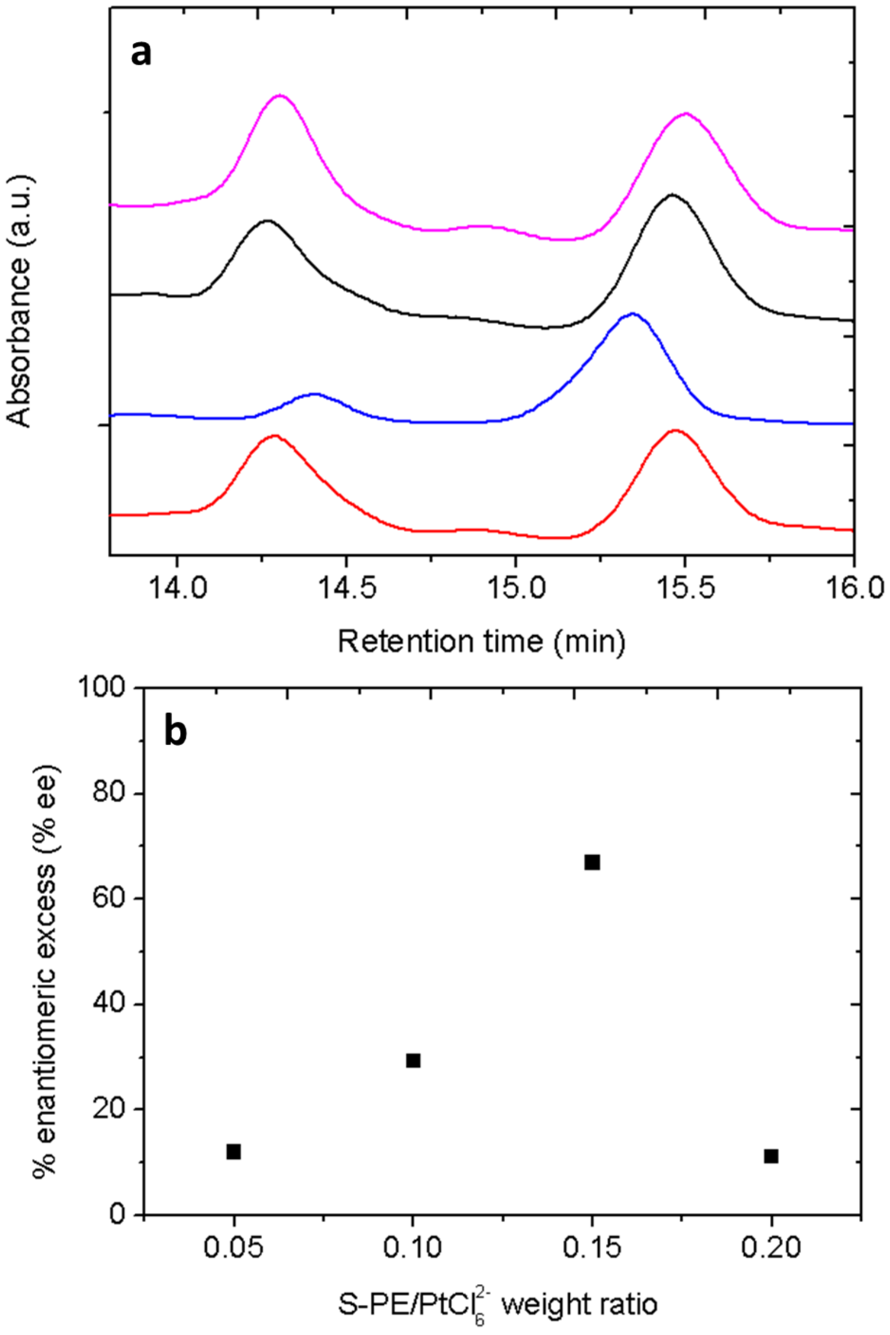

Supplementary Figure 7. Monitoring the effect of imprinting ratio on enantioselectivity. (a) HPLC chromatograms of the electrosynthesis products obtained at -0.45 V with a pulse time of 10s by electrodes imprinted with (*S*)-PE with various (*S*)-PE/$PtCl_6^{2-}$ weight ratios; 0.05 (pink), 0.10 (black), 0.15 (blue), and 0.20 (red). (b) % enantiomeric excess as a function of chiral template to metal salt ratio.

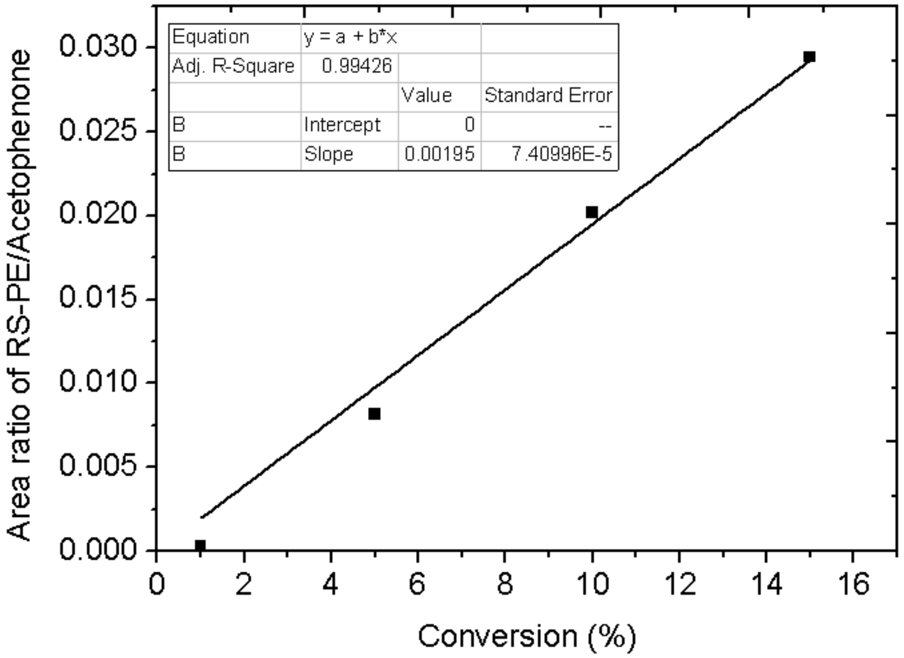

Supplementary Figure 8. HPLC calibration curve as a function of the percentage of overall conversion. For this calibration curve, the HPLC peak area ratios of 1-phenylethanol to acetophenone were measured. Mixtures with various ratios of 1-phenylethanol to acetophenone were prepared to represent different degrees of educt conversion in the range of 0-15% and were analyzed by HPLC. HPLC was performed on a Shimadzu LC-2030C3D equipped with a chiral HPLC column (CHIRALPAK IB, 250mm x 4.6mm inner diameter) using a mobile phase containing 95% heptane/5% i-propanol at a flow rate of 0.5 ml. min^{-1} and a detection at 215 nm. Based on this calibration curve it is possible to estimate the overall conversion for the present experiments (using a relaxation time of 120s) to be in the 10% range when comparing the ratio of the acetophenone and phenylethanol HPLC signals (see **Supplementary Figure 9**).

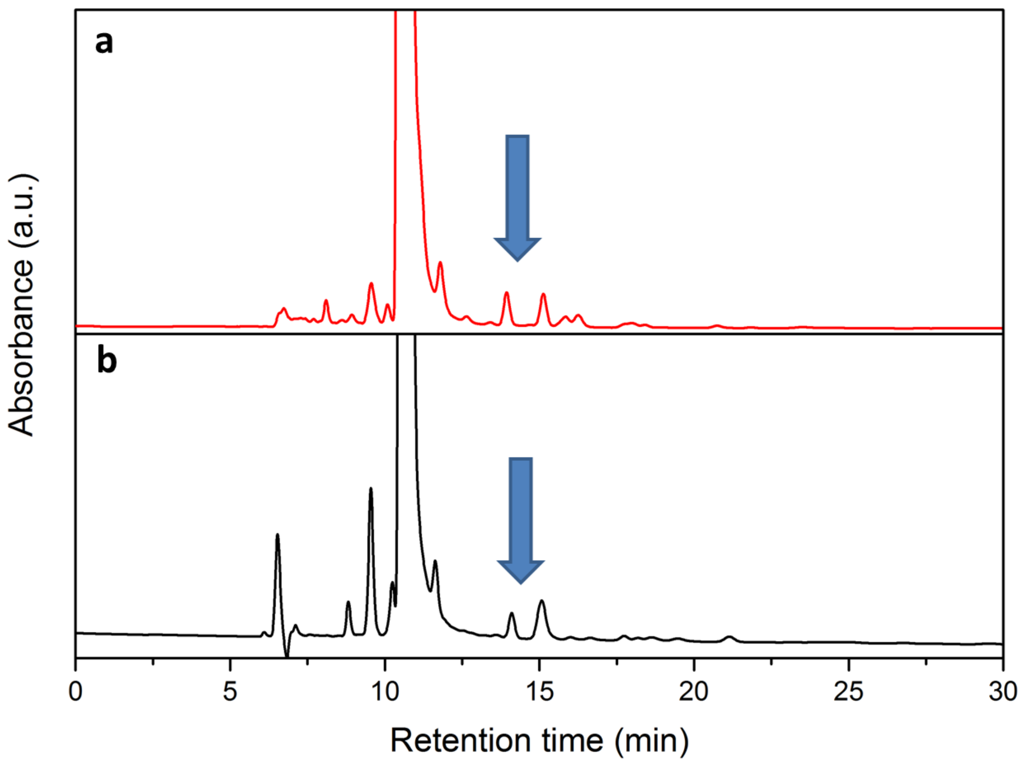

Supplementary Figure 9. Full HPLC chromatogram of a standard mixture of acetophenone and racemic phenylethanol (a) and the electrosynthesized mixture (b). The retention time domaine for the phenylethanol enantiomers is indicated by the blue arrow. Acetophenone is the starting compound and constitutes the main peak at t=11min. Parasitic peaks in (b) are not originating from impurities produced during the electrosynthesis because they are already present in the standard mixture of commercial acetophenone and racemic phenyethanol as can be seen from (a).

Supplementary Tables

Entry	Electrodes	PE/$PtCl_6^{2-}$ weight ratio	Reduction potential (V)	Charge density ($C.cm^{-2}$)	% Enantiomeric excess
1	CIMP imprinted with R-PE	0.05	-0.50	4	5.8
2	CIMP imprinted with R-PE	0.05	-0.45	4	6.6
3	CIMP imprinted with R-PE	0.10	-0.45	4	8.6
4	CIMP imprinted with R-PE	0.15	-0.45	4	11.1
5	CIMP imprinted with S-PE	0.10	-0.45	4	-9.1
6	CIMP imprinted with S-PE	0.10	-0.45	8	-12.2
7	CIMP imprinted with S-PE	0.15	-0.45	8	-13.3

Supplementary Table 1. Effect of the reduction potential, PE/$PtCl_6^{2-}$ weight ratio, and metal deposition charge density (porous metal layer thickness) on the enantioselectivity of acetophenone reduction obtained for conventional steady-state electroreduction on CIMP. Enantiomeric excess (% ee) = (RPE-SPE)/(RPE+SPE)x100, where RPE and SPE are the HPLC peak areas of (*R*)-PE and (*S*)-PE, respectively. The s.e.m. is ± 0.98% ee calculated from a set of three identical experiments.

Entry	Electrodes	$PE/PtCl_6^{2-}$ weight ratio	Relaxation time (s)	Pulse time (s)	% Enantiomeric excess
1	CIMP imprinted with S-PE	0.15	120	2	-90.7
2	CIMP imprinted with S-PE	0.15	120	10	-66.9
3	CIMP imprinted with S-PE	0.15	120	30	-58.4
4	CIMP imprinted with S-PE	0.15	120	60	-36.2
5	CIMP imprinted with R-PE	0.15	120	10	+60.4
6	CIMP imprinted with S-PE	0.05	120	10	-12.0
7	CIMP imprinted with S-PE	0.10	120	10	-29.3
8	CIMP imprinted with S-PE	0.20	120	10	-11.2

Supplementary Table 2. Enantioselective synthesis of 1-phenylphenol using chiral imprinted mesoporous platinum for pulsed electroreduction with a reduction potential and metal deposition charge density of -0.45 V and 8 $C.cm^{-2}$, respectively.
Enantiomeric excess (% ee) = (RPE-SPE)/(RPE+SPE)x100, where RPE and SPE are the HPLC peak areas of (*R*)-PE and (*S*)-PE, respectively.

Experiments	% Enantiomeric excess
1st Run	-66.9
2nd Run	-45.3
3rd Run	-39.0

Supplementary Table 3. Enantioselective synthesis of 1-phenylethanol using the same chiral imprinted mesoporous platinum (metal deposition charge density of 8 $C.cm^{-2}$, electrode was imprinted with a (*S)*-PE/$PtCl_6^{2-}$ weight ratio of 0.15) several times for pulsed electroreduction of acetophenone (reduction potential -0.45 V, relaxation time 120 s, pulse time 10 s).
